# MicroRNA-370 carried by M2 macrophage-derived exosomes alleviates asthma progression through inhibiting the FGF1/MAPK/STAT1 axis

**DOI:** 10.7150/ijbs.59715

**Published:** 2021-04-23

**Authors:** Chunlu Li, Chengsi Deng, Tingting Zhou, Jiapeng Hu, Bing Dai, Fei Yi, Na Tian, Lijun Jiang, Xiang Dong, Qingfeng Zhu, Siyi Zhang, Hongyan Cui, Liu Cao, Yunxiao Shang

**Affiliations:** 1 Department of Pediatrics, Shengjing Hospital of China Medical University, Shenyang 110004, China.; 2 College of Basic Medicine Science, China Medical University, Shenyang 110122, China.; 3 Jilin Tuohua Biotechnology Co., Ltd. Changchun, Jilin 13000, China.

**Keywords:** M2 macrophages, exosomes, miR-370, FGF1, asthma

## Abstract

Emerging evidence has suggested the functions of exosomes in allergic diseases including asthma. By using a mouse model with asthma induced by ovalbumin (OVA), we explored the roles of M2 macrophage-derived exosomes (M2Φ-Exos) in asthma progression. M2Φ-Exos significantly alleviated OVA-induced fibrosis and inflammatory responses in mouse lung tissues, as well as inhibited abnormal proliferation, invasion, and fibrosis-related protein production in platelet derived growth factor (PDGF-BB) treated primary mouse airway smooth muscle cells (ASMCs). The OVA administration in mice or the PDGF-BB treatment in ASMCs reduced the expression of miR-370, which was detected in M2Φ-Exos by miRNA sequencing. However, treating the mice or ASMCs with M2Φ-Exos reversed the inhibitory effect of OVA or PDGF-BB on miR-370 expression. We identified that the target of miR-370 was fibroblast growth factor 1 (*FGF1*). Downregulation of miR-370 by Lv-miR-370 inhibitor or overexpression of FGF1 by Lv-FGF1 blocked the protective roles of M2Φ-Exos in asthma-like mouse and cell models. M2Φ-Exos were found to inactivate the MAPK signaling pathway, which was recovered by miR-370 inhibition or FGF1 overexpression. Collectively, we conclude that M2Φ-Exos carry miR-370 to alleviate asthma progression through downregulating FGF1 expression and the MAPK/STAT1 signaling pathway. Our study may offer a novel insight into asthma treatment.

## Introduction

Affecting over 300 million people, asthma is a chronic respiratory disease with rising global incidences that loads individuals and the healthcare system with high economic costs during the long disease courses 1,2. Asthma is a heterogeneous disease with genetics and environmental exposure reported as the main contributors 3. Asthma is featured with airway inflammation, variable airflow obstruction, mucous hypersecretion, airway hyperresponsiveness (AHR), and airway remodeling, which involves an increase in the number of goblet cells, epithelial damage, subepithelial fibrosis, an increase in airway smooth muscles and vascularity 4,5. Despite significant improvements in medicine and fundamental research, asthma remains a burden for children and adults and leads to considerable morbidity 3. Conventional drugs are effective for patients with mild to moderate asthma but are less useful for those with severe asthma 2. The discovery of novel molecules involved in asthma progression will shed light on more precise approaches for asthma treatment.

Exosomes, a class of small extracellular vesicles (30-100 nm in diameter) that enable intercellular communication by carrying different molecules including nucleic acids [DNA, mRNA, and microRNAs (miRNAs)], proteins, and lipids, are implicated in the cell functions and pathology of multiple diseases including asthma 6. Macrophage-derived exosomes account for a large part of circulating micro-vesicles in blood 7. Macrophages can polarize into two major types depending on the stimuli: the pro-inflammatory M1 type and the anti-inflammatory M2 type 8. miRNAs are a major class of exosome cargo that play crucial roles in inflammation, tissue repair, and fibrogenesis 9. They are also involved in epithelial differentiation, inflammation mucus production, and airway remodeling in asthma 10. A recent study noted that M2Φ-Exos carried miR-148a to reduce inflammation and alleviate myocardial ischemia/reperfusion injury 11. However, the functions of M2Φ-Exos in asthma progression as well as the molecules involved have not been concerned. In the present study, miR-370 was identified as an obviously upregulated miRNA by M2Φ-Exos in an asthma mouse or cell model according to an RT-qPCR result. miR-370 has been reported as an RNA sponge for long non-coding RNA (lncRNA) XIST to reduce cell apoptosis and inflammation injury in acute pneumonia 12. But its role in lung injury in asthma remains unknown. A primary function of miRNAs is to regulate downstream mRNA expression through direct binding to the 3' untranslated region (3'-UTR) of target transcripts 13. Our integrated bioinformatics analyses suggested fibroblast growth factor 1 (*FGF1*) mRNA as a direct target of miR-370. FGF1 is a member of the FGF family that plays diverse functions in development, wound healing, angiogenesis, neurogenesis, and metabolism 14,15. It was found highly expressed in idiopathic pulmonary fibrosis and was related to wound healing and fibrosis 16. Herein, this study was carried out to explore whether M2Φ-exosomal miR-370 alleviated asthma progression via inhibiting the expression of FGF1, and an OVA-induced mouse model and PDGF-treated ASMCs model were used to validate this hypothesis.

## Materials and methods

### Exaction and culture of macrophages

C57BL/6 J mice purchased from the Beijing Charles River company were used for this study. In brief, the bone marrow cells extracted from mice were cultured in high-glucose Dulbecco's modified medium (DMEM, Gibco, 12100046) supplemented with 10% fetal bovine serum (FBS, Gibco,10099141), 1% penicillin-streptomycin (Gibco, SV30010), and 20 ng/mL macrophage colony-stimulating factor (M-CSF, Sigma, SRP3221). Next, the BMDMs were cultured in 20 ng/mL interleukin-4 (IL-4, Sigma, SRP3211) for M2 polarization. The M0 and M2 macrophages were harvested for flow cytometry analysis.

### Extraction and identification of exosomes

After 72 hours of culture, collect the supernatant of M2 macrophage culture dish. The samples were centrifuged at 1000 g for 10 minutes to discard cell debris and dead cells. The supernatant was collected by centrifuging at 10,000 g at 4 °C for 30 minutes to remove shed micro-vesicles. Then, the samples were resuspended in culture medium and ultra-centrifuged at 100,000 g at 4 °C for 70 minutes, washed in phosphate buffer saline (PBS), and centrifuged at 100,000 for another 30 minutes. The precipitates containing exosomes were resuspended in PBS. A transmission electron microscope (Thermo Fisher) was used for morphology evaluation. A Nanosizer instrument (Nano FCM) was used for dynamic light scattering analysis.

### Establishment of a mouse model with asthma

Male BALB/c mice (6-8 weeks old, 18-24 g) were treated with ovalbumin (OVA, Sigma, A5503) to induce asthma. In brief, 20 g complete Freund' s adjuvant was emulsified in 1mg aluminum hydroxide (Sigma, 239186), and then administrated into each mouse at a total volume of 0.2 mL on the 0, 7^th^, and 14^th^ day through intraperitoneal injection to sensitize the mice to OVA. On the 15^th^ day, each mouse was exposed to 1% OVA aerosol for 1 hour for 7 days. Mice in the control group were treated with normal saline instead. In addition, mice subjected to M2Φ-Exos treatment were treated with 20 μg M2Φ-Exos for each mouse since the 20^th^ day for continuous 3 days. This study was approved by the Ethics Committee of China Medical University.

### Hematoxylin and eosin (HE) staining

The left lung tissues were fixed in 4% paraformaldehyde overnight. The tissues were embedded in paraffin, cut into 4 μm sections, dewaxed, and stained with hematoxylin and eosin (Sigma) to observe the histological changes and cell infiltration into the peribronchial connective tissues.

### Masson's trichrome staining

The above paraffin-embedded sections were successively dewaxed, stained with Weigert solution (Sigma) for 5 minutes, fully washed, treated with Ponceau acid fuchsin for 5 minutes, soaked in 2% acetic acid aqueous solution for 1 minute, and then differentiated in 1% aqueous phosphomolybdic acid for 3 minutes Next, the sections were treated with aniline blue for 5 minutes soaked in 0.2% acetic acid aqueous solution for 30 seconds, permeated using xylene and sealed with neutral resin.

### Terminal deoxynucleotidyl transferase (TdT)-mediated dUTP nick end labeling (TUNEL)

An *in-situ* apoptosis detection kit and a 3,3-diaminobenzidine (DAB) kit (Roche) were used for TUNEL analysis. In brief, the paraffin-embedded sections were dewaxed and permeabilized and then treated with a TUNEL reaction mixture for a 60 minutes warm incubation at 37 °C. Then the sections were further treated with 50 μL POD for incubation of 30 minutes. After three times of PBS washes, the sections were incubated with 50 μL DAB substrates for 5 minutes. The nuclei of cells were stained by hematoxylin, and the sections were sealed by cover glasses.

### BALF analysis

The number of total blood cells in BALF was determined using a hemacytometer. Differences in cell number of neutrophilic granulocytes, eosinophilic granulocytes, and lymphocytes were evaluated from at least 200 cells. The BALF was centrifuged at 300 g at 4 °C for 15 minutes to collect the supernatant. Levels of monocyte chemotactic protein-1 (MCP-1), interleukin (IL)-1β, IL-6, and tumor necrosis factor-α (TNF-α) in cell-free BALF were determined using Elisa kits (Biolegend).

### AHR measurement

Mice were anesthetized by 1% pentobarbital sodium and subjected to mechanical ventilation, and the AHR of mice was measured using an animal pulmonary function instrument (Buxco Electronics). In brief, an ascending series of methacholine (3.125-50 mg/mL) was administrated into the trachea through the connected atomizer. The baseline airway resistance was evaluated by atomized PBS. The resistance index (RI) of the total lung and airway was determined as per the protocols of the instrument.

### Immunohistochemical (IHC) staining

The expressions of p-MAPK and p-STAT1 were determined by IHC staining. The paraffin-embedded sections were dewaxed, hydrated in alcohol, and treated with citric acid for antigen retrieval. Then, the sections were exposed to endogenous POD blocker for 10 minutes and incubated with the primary antibodies against p-MAPK (CST, 4370) and p-STAT1(CST, 9167) at 4 °C overnight, and then with the secondary antibody horseradish peroxidase (HRP)-labeled goat anti-rabbit immunoglobulin G at room temperature for 30 minutes and exposed to DAB for 5 minutes. Finally, the sections were stained with hematoxylin for microscope observation.

### Airway smooth muscle cells culture

Airway smooth muscle cells (ASMCs) from mouse bronchia were cultured in RPMI-1640 medium containing 10% FBS and 100 units/ml of penicillin/streptomycin at 37 °C with 5% CO^2^. The cells were treated with 50 ng/mL PDGF-BB. PDGF-BB has been reported to abundantly exist in asthma patients, and it can trigger tracheal remodeling induced by ASMC proliferation 17-21.

### Colony formation assay

In brief, the untreated ASMCs were sorted into 6-wells culture plates (5000 cells per well) and cultured at 37 °C overnight. Next, the cells after different treatments were loaded on culture dishes for 48 hours, and then the cells were further cultivated in normal medium for 5 days. After that, the cell colonies were fixed in 4% paraformaldehyde, stained with Coomassie blue R-250 stain solution, and observed under the microscope to evaluate the number of colonies formed.

### Transwell assay

Migration of cells was determined using Transwell chambers (Corning Costar, 24-well inserts, diameter, 8 μm). In brief, cells resuspended in 200 μl serum-free medium were loaded in the apical chambers, while the basolateral chambers were filled with 20% FBS-supplemented medium. After 24 hours of incubation at 37 °C, the chambers were taken out. The cells on the upper surface of the membranes were removed using cotton swabs, and the migrated cells on the lower surface were fixed in 75% ethanol, stained with hematoxylin, and observed under a microscope with five random fields included. Cell invasion was measured in a similar manner with the apical chambers precoated with Matrigel before cell loading.

### Reverse transcription quantitative polymerase chain reaction (RT-qPCR)

Total RNA from tissue and cells was extracted using a TRIzol reagent (Takara). Reverse transcription was performed using PrimeScript RT reagent Kit with gDNA Eraser (Takara). The expression levels of miR-370 and U6 were determined using the Bulge-Loop miRNA qRT-PCR Starter Kit (RiboBio), with U6 as the internal control. The expression levels of FGF1, Fibronectin, Vimentin, and GAPDH were determined using TB Green Premix Ex Taq II (Takara), with GAPDH as the internal control. The PCR performed on Roche LightCycler 480II and analyzed by the related software. The primers are listed in Supplementary Table S1.

### Immunofluorescence staining

The ASMCs were fixed in 4% paraformaldehyde for 1 hour at 4 °C, blocked in 20% goat serum for 2 hours at 4 °C, and incubated with the antibodies against α-smooth muscle actin (α-SMA, Santa Cruz, sc-53142), Fibronectin(Santa Cruz, sc-8422), Vimentin (Santa Cruz, sc-6260), and p-MAPK (CST, 4370) at 4 °C overnight, and then with fluorescein isothiocyanate-labeled secondary antibody (Invitrogen Alexa Fluor, A21203, A21207) at room temperature for 1 hour. In addition, DAPI (Thermo Fisher, 62247) was used to stain the nuclei. The staining was observed under an inverted fluorescence microscope (Nikon).

### Western blot analysis

The right lung tissues were cut into 2-3 mm^3^ debris and prepared as tissue homogenate by a handheld homogenizer. The protein concentration was determined using the bicinchoninic acid method. Then, an equal volume of protein was run on 5-12% SDS PAGE electrophoresis and transferred on PVDF membranes. After being blocked by 5% BSA, the membranes were incubated with the primary antibodies to α-tubulin (Sigma, T6199), CD63 (Abcam, ab217345), CD9 (Abcam, ab92726), TSG101 (Thermo Fisher, PA5-82236), Calnexin (Immunoway, YP0041), FGF1 (CUSABIO), MAPK (CST, 8690), p-MAPK (CST, 4370), STAT1 (CST, 9172), and p-STAT1 (CST, 9167) at 4 °C overnight, and then with HRP-conjugated secondary antibodies at room temperature for 1 hour. The bands were detected by enhanced chemiluminescence detection kit (Thermo Fisher, 32106) and visualized via the DNR western blot detection system.

### Dual-luciferase reporter gene assay

The putative binding site between miR-370 and the 3'-UTR of FGF1 was predicted on TargetScan (http://www.targetsc an.org/vert_72/). Next, the wild-type (WT) or mutant type (MT) FGF1 3'-UTR sequences were constructed, amplified, and cloned to the pLUC luciferase reporter vectors or vector controls (Promega, Corp., Madison, Wisconsin, USA) to establish FGF1-WT and FGF1-MT vectors. The vectors were co-transfected with either miR-370 mimic or negative control (NC) mimic into 293T cells in strict accordance with a Lipofectamine 2000 kit (Invitrogen). Forty-eight hours later, the cells were collected and lysed for firefly luciferase activity detection using the corresponding assay kit (GeneCopoeia, Rockville).

### Data analysis

All statistical analyses were performed on the software GraphPad Prism version 7.0. Data were expressed as mean standard deviation (SD) from no less than three independent experiments. Differences were compared by the t-test (two groups) and one-way or two-way analysis of variance (ANOVA, more than two groups). Differences were considered significant by a *p*-value less than 0.05 *(p* < 0.05).

## Results

### M2Φ-Exos inhibit OVA-induced lung injury and inflammatory cytokine secretion in mice

**Figure 1:**
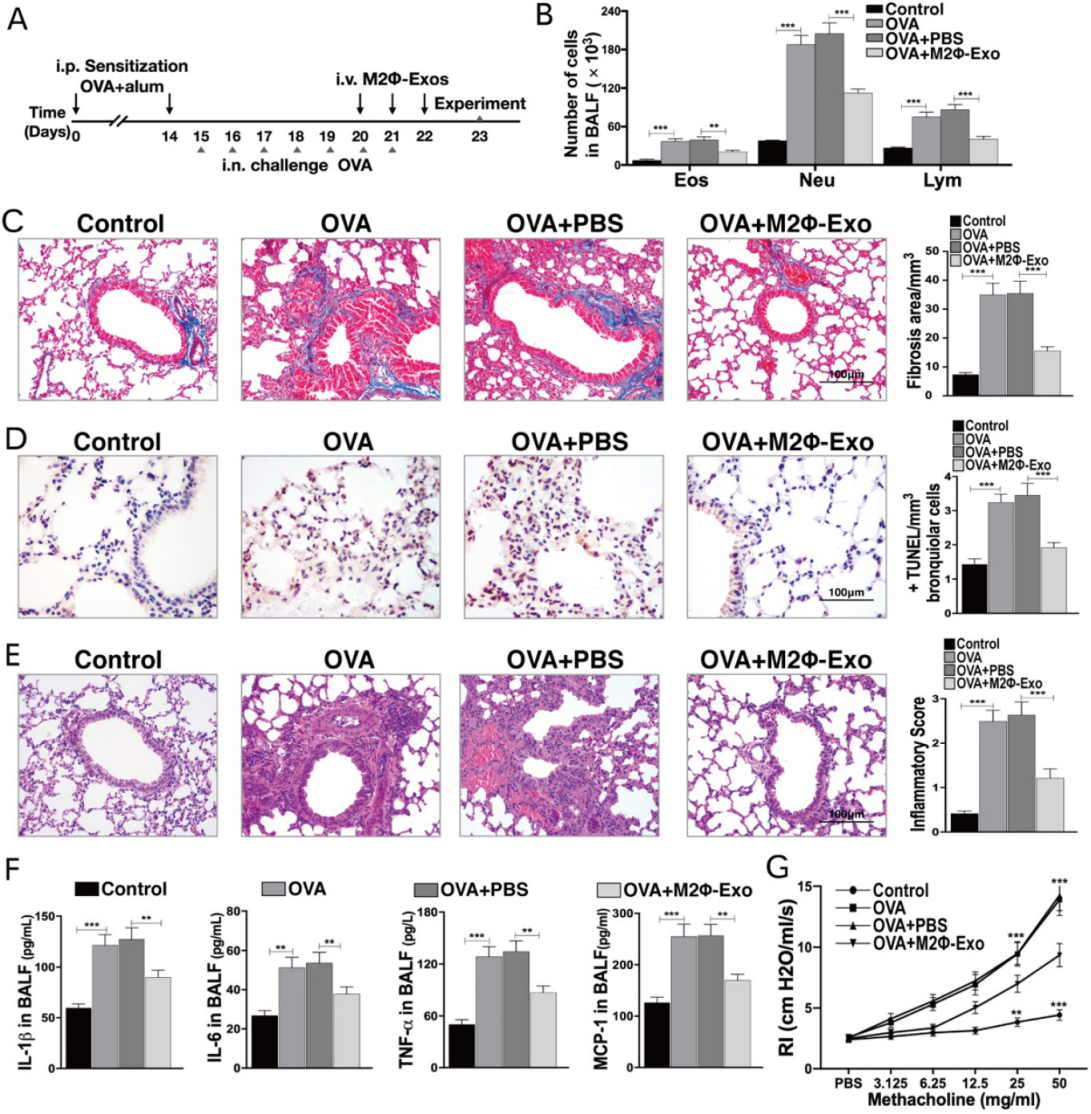
** M2Φ-Exos inhibit OVA-induced lung injury and inflammatory cytokine secretion in mice**. (A) Complete Freund's adjuvant was used to sensitize the mice to OVA, and the mice were treated with 1% OVA aerosol on the 15^th^ day for continuous 7 days, with the administration of M2Φ-Exos on the 20^th^ day for continuous 3 days. (B) Measurement of the number of eosinophilic granulocytes, neutrophil granulocytes and lymphocytes in BALF. (C) Fibrosis in mouse lung tissues measured by Masson's trichrome staining. (D) Apoptosis of cells determined by TUNEL staining. (E) Pathological changes in mouse lung tissues measured by HE staining. (F) Protein levels of IL-1β, IL-6, TNF-α, and MCP-1 in BALF measured by ELISA kits. (G) AHR determined by lung resistance. In each group, n = 5. Data were expressed as mean SD. In panels C, D, E and F, data were analyzed by one-way ANOVA while data in panels B and G by two-way ANOVA, followed by Tukey's multiple comparison test. **p < 0.01, ***p <0.001.

Polarization of M2 macrophages and extraction of exosomes were shown in Fig S1. The OVA was used to induce asthma-like symptoms in mice, and the procedures were presented in Fig. 1A. After OVA treatment, it was found that the number of eosinophilic granulocytes, neutrophil granulocytes, and lymphocytes were increased (Fig. 1B). In addition, it was found that the fibrosis (Fig. 1C) and cell apoptosis (Fig. 1D) in OVA-treated mouse lung tissues were notably increased. The HE staining showed significant inflammatory cell infiltration in mouse lung tissues after OVA treatment (Fig. 1E). And the increase in the levels of inflammatory cytokines IL-1β, IL-6, TNF-α, and MCP-1 was detected by the ELISA assay (Fig. 1F). Besides, the RI of OVA-treated mice after methacholine treatment was apparently increased (Fig. 1G). Subsequently, the OVA-induced asthma mice were treated with M2Φ-Exos, and we found a significant inhibition in fibrosis and cell apoptosis in mouse lung tissues, a reduction in the number of granulocytes, and a decline in the secretion of OVA-induced IL-1β, IL-6, TNF-α, and MCP-1 (Fig. 1B-F).

### M2Φ-Exos inhibit proliferation and inflammation in PDGF-BB treated ASMCs

**Figure 2:**
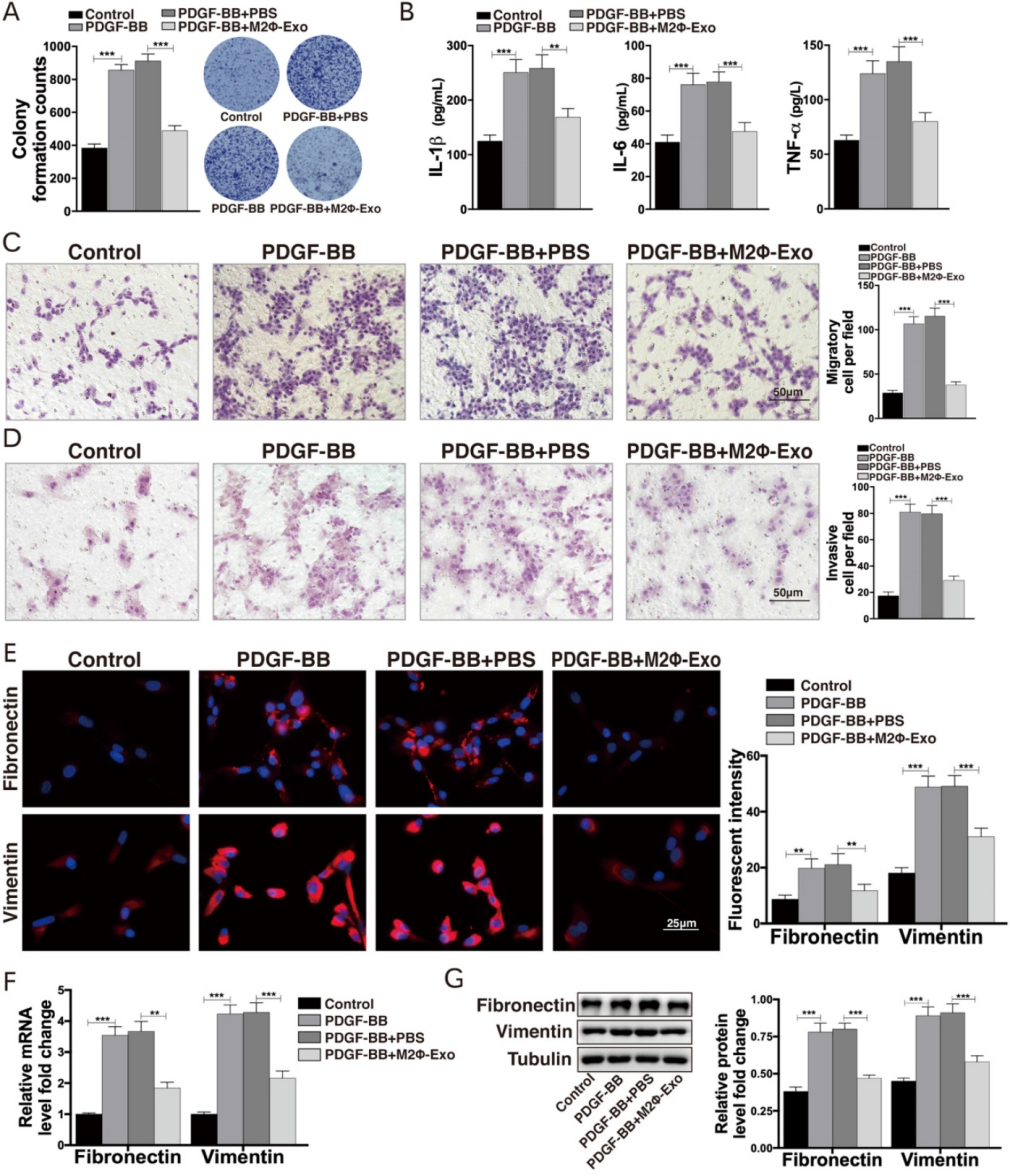
** M2Φ-Exos inhibit proliferation and inflammation in ASMCs induced by PDGF-BB.** (A) Number of cell colonies measured by colony formation assay. (B) Secretion of IL-1β, IL-6 and TNF-α in ASMCs determined by ELISA kits. (C) Migration and (D) invasion abilities of ASMCs measured by Transwell assays. (E) Expression of fibronectin and vimentin in ASMCs determined by immunofluorescence staining. (F) Expression of mRNA of fibronectin and vimentin in ASMCs determined by RT-qPCR. (G) Expression of fibronectin and vimentin in ASMCs determined by Western bolt. Data were expressed as mean SD from at least three independent experiments. In panels A, B, C and D, data were analyzed by one-way ANOVA, while data in panels E, F and G were analyzed by two-way ANOVA, followed by Tukey's multiple comparison test. **p < 0.01, ***p < 0.001.

ASMCs were treated with PDGF-BB, after which the proliferation of ASMCs and the production of IL-1β, IL-6 and TNF-α in cells were notably increased (Fig. 2A,2B). In addition, the Transwell assays suggested that the migration and invasion abilities of ASMCs were enhanced after PDGF-BB treatment (Fig. 2C,2D). Next, the expressions of the fibrosis-related factors fibronectin and vimentin were measured by immunofluorescence staining, RT-qPCR, and Western blot. It was found that the expressions of fibronectin and vimentin were obviously increased after PDGF-BB treatment (Fig. 2E-G). Then, we treated the PDGF-BB-induced ASMCs with M2Φ-Exos, after that the proliferation of ASMCs, the secretion of IL-1β, IL-6 and TNF-α, the number of migrated and invaded cells, and the fibrosis-related factors fibronectin and vimentin were significantly reduced (Fig. 2A-G).

### M2Φ-Exos carry miR-370 to suppress FGF1 expression

**Figure 3:**
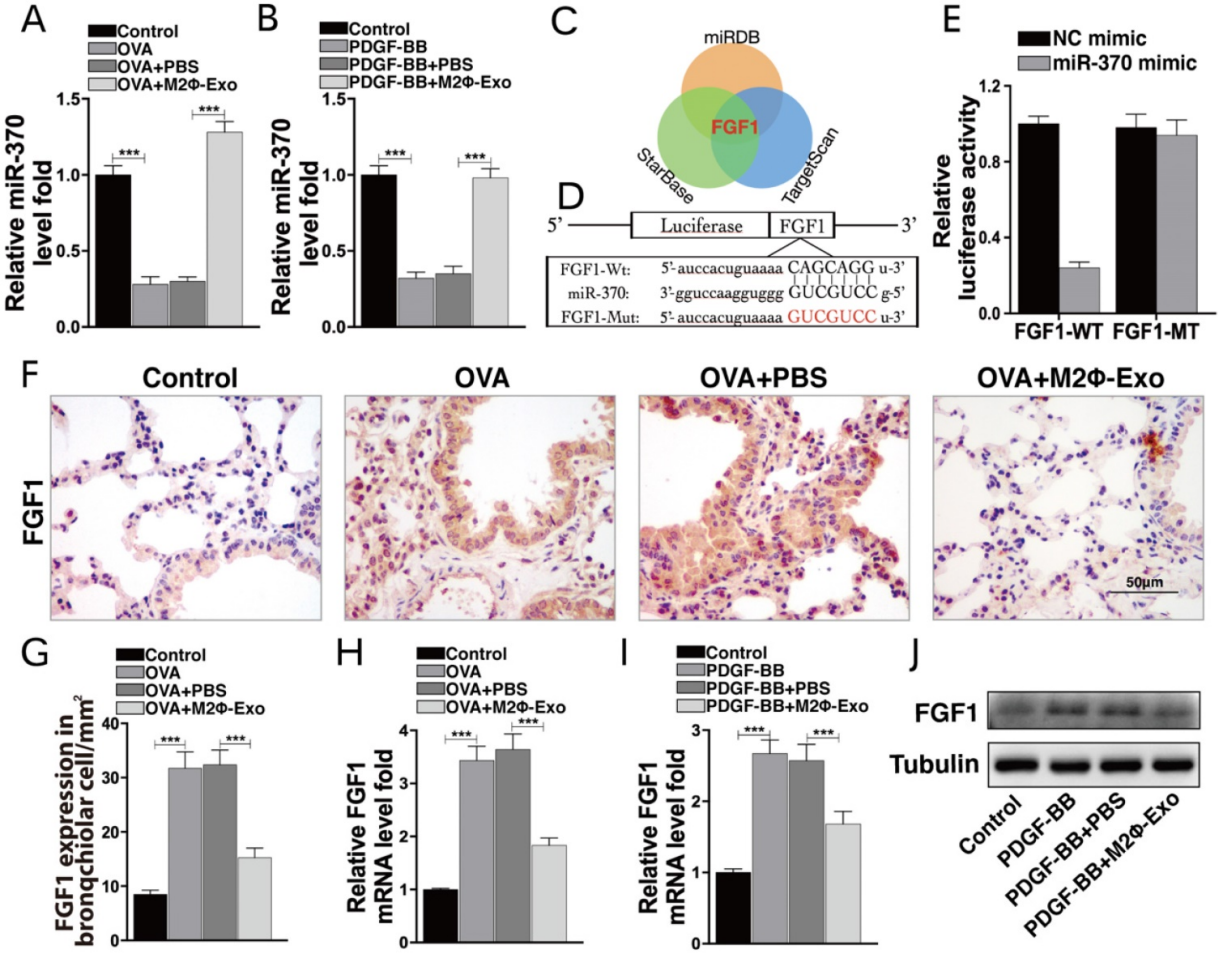
** M2Φ-Exos carry miR-370 to suppress FGF1 expression**. miR-370 expression in (A) mouse lung tissues and (B) ASMCs measured by RT-qPCR. (C) Targeting mRNAs of miR-370 predicted on TargetScan, miRDB and StarBase. (D) Predicted miR-370 binding site in FGF1 (FGF1-WT) and mutant sequence (FGF1-MT). (E) Binding relationship between miR-370 and FGF1 validated through a dual luciferase assay. (F-G) FGF1 expression in mouse lung tissues determined by IHC staining. (H) Expression of mRNA of FGF1 in mouse lung tissues determined by RT-qPCR. mRNA (I) and protein (J) expression of FGF1 in ASMCs determined by RT-qPCR and Western blot, respectively. Data were expressed as mean SD from at least three independent experiments. In panels A, B, G, H and I, data were analyzed by one-way ANOVA, while data in panel E were analyzed by two-way ANOVA, followed by Tukey's multiple comparison test. **p < 0.01, ***p <0.001.

As M2Φ-Exos may carry miRNAs to exert their functions, we then analyzed all miRNAs from M2-Exos using miRNA-seq. Among them, miR-370 had aroused our attention. miR-370 has been documented to suppress LPS-induced lung injury 12,22, and thus was selected as the subject of the following experiments. We first determined miR-370 expression in mouse lung tissues using RT-qPCR. The results showed that miR-370 was downregulated in OVA-induced mice but then recovered after M2Φ-Exos treatment (Fig. 3A). A similar trend was found in PDGF-BB-treated ASMCs models. miR-370 was downregulated in ASMCs after PDGF-BB treatment but then upregulated after M2Φ-Exos administration (Fig. 3B). Thereafter, we focused on the target mRNAs of miR-370. The integrated analysis on three bioinformatics systems TargetScan (http://www.targetscan.org/vert_72/), miRDB (http://mirdb.org/) and StarBase (http://starbase.sysu.edu.cn/) predicted FGF1 as an intersected outcome of miR-370 (Fig. 3C). According to miR-370 predicted binding site in FGF1, we designed pLUC-FGF1-WT vector and pLUC-FGF1-MT vector (Fig. 3D). To validate the binding relationship between miR-370 and the 3'-UTR of FGF1 mRNA, dual-luciferase reporter gene assay was performed where we found that the luciferase activity in 293T cells co-transfected with miR-370 mimic and pLUC-FGF1-WT vector was obviously decreased, while that in cells transfected with either NC mimic or pLUC-FGF1-MT vector showed no major changes (Fig. 3E). An IHC staining of FGF1 in mouse lung tissues was additionally performed. It was found that the FGF1 expression was increased after OVA treatment but inhibited by M2Φ-Exos (Fig. 3F-H). Besides, a similar result was found in ASMCs (Fig. 3I,3J).

### Lv-FGF1 or Lv-miR-370 inhibitor blocks the protective roles of M2Φ-Exos in OVA-treated mice

**Figure 4:**
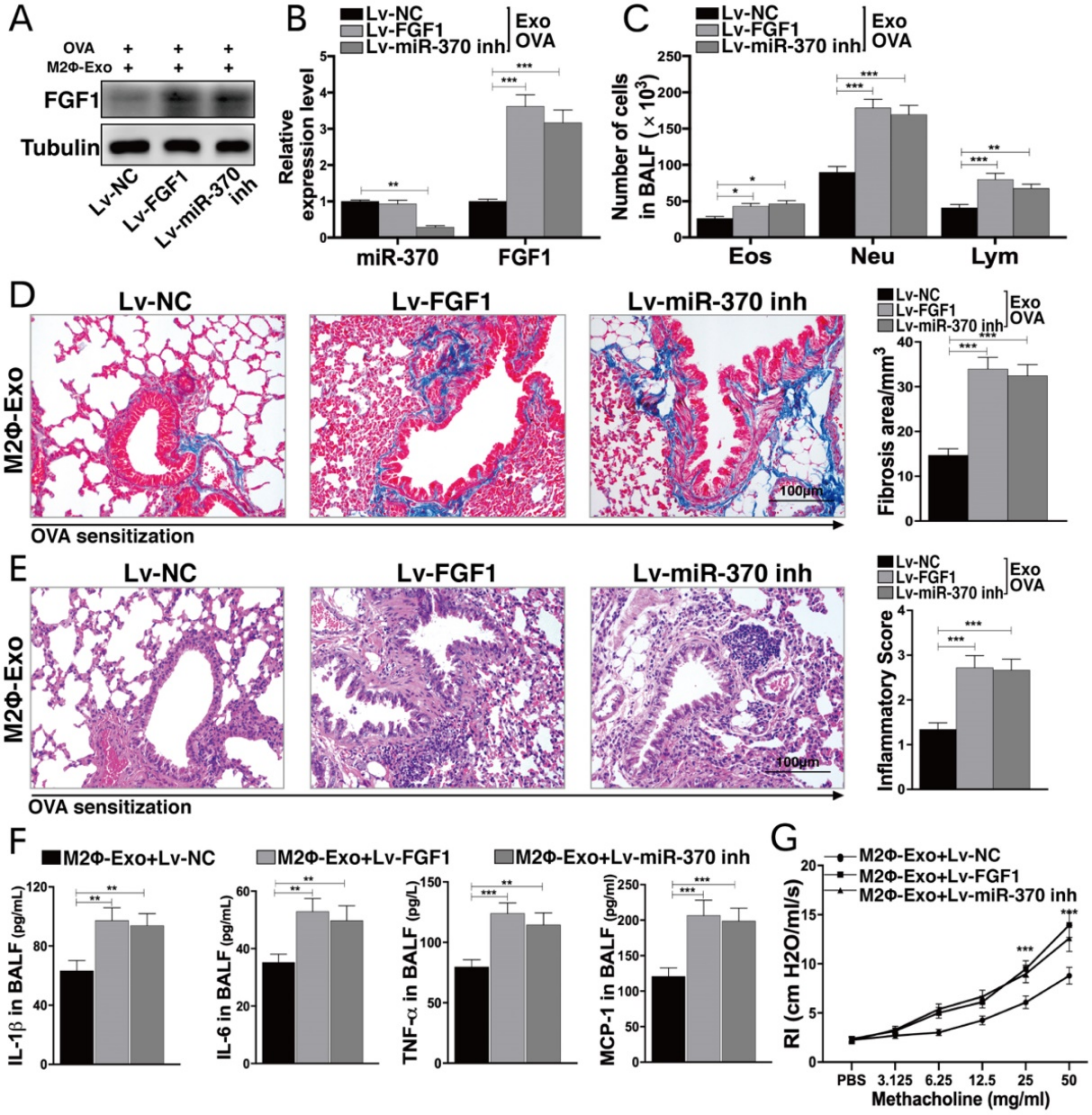
** Lv-FGF1 or Lv-miR-370 inhibitor blocks the protective roles of M2Φ-Exos in OVA-treated mice**. Lv-FGF1 or Lv-miR-370 inhibitor was further administrated in OVA- and M2Φ-Exos-treated mice, (A) the protein expression of FGF1 and (B) miR-370 and FGF1 mRNA were determined by Western blot and RT-qPCR, respectively. (C) Measurement of the number of eosinophilic granulocytes, neutrophil granulocytes and lymphocytes in BALF. (D) Fibrosis in mouse lung tissues determined by Masson's trichrome staining. (E) Pathological changes in mouse tissues determined by HE staining. (F) Expression of IL-1β, IL-6, TNF-α and MCP-1 in BALF determined by ELISA kits. (G) AHR determined by lung resistance. In each group, n = 5. Data were expressed as mean SD. In panels B, D, E and F, data were analyzed by one-way ANOVA while data in panels C and G were determined by two-way ANOVA, followed by Tukey's multiple comparison test. **p < 0.01, ***p < 0.001.

To validate the roles of FGF1 and miR-370 in lung injury, the mice were infected with lentiviral vectors (Lv)-FGF1 or Lv-miR-370 inhibitor at a concentration of 2×10^9^ PFU/ml, and the expression efficacy was determined by Western blot and RT-qPCR (Fig. 4A,4B). In OVA-induced mice, it was found that the number of granulocytes in BALF was notably increased after Lv-FGF1 or Lv-miR-370 inhibitor treatment (Fig. 4C). Thereafter, we noticed that the fibrosis and inflammation in mouse lung tissues suppressed by M2Φ-Exos were increased by the further upregulation of FGF1 or miR-370 inhibition (Fig. 4D,4E). In addition, the concentrations of IL-1β, IL-6, TNF-α, and MCP-1 in BALF were increased (Fig. 4F), and the RI in mice after methacholine treatment was increased as well (Fig. 4G).

### Lv-FGF1 or Lv-miR-370 inhibitor inhibits the protective roles of M2Φ-Exos in PDGF-BB treated ASMCs

**Figure 5:**
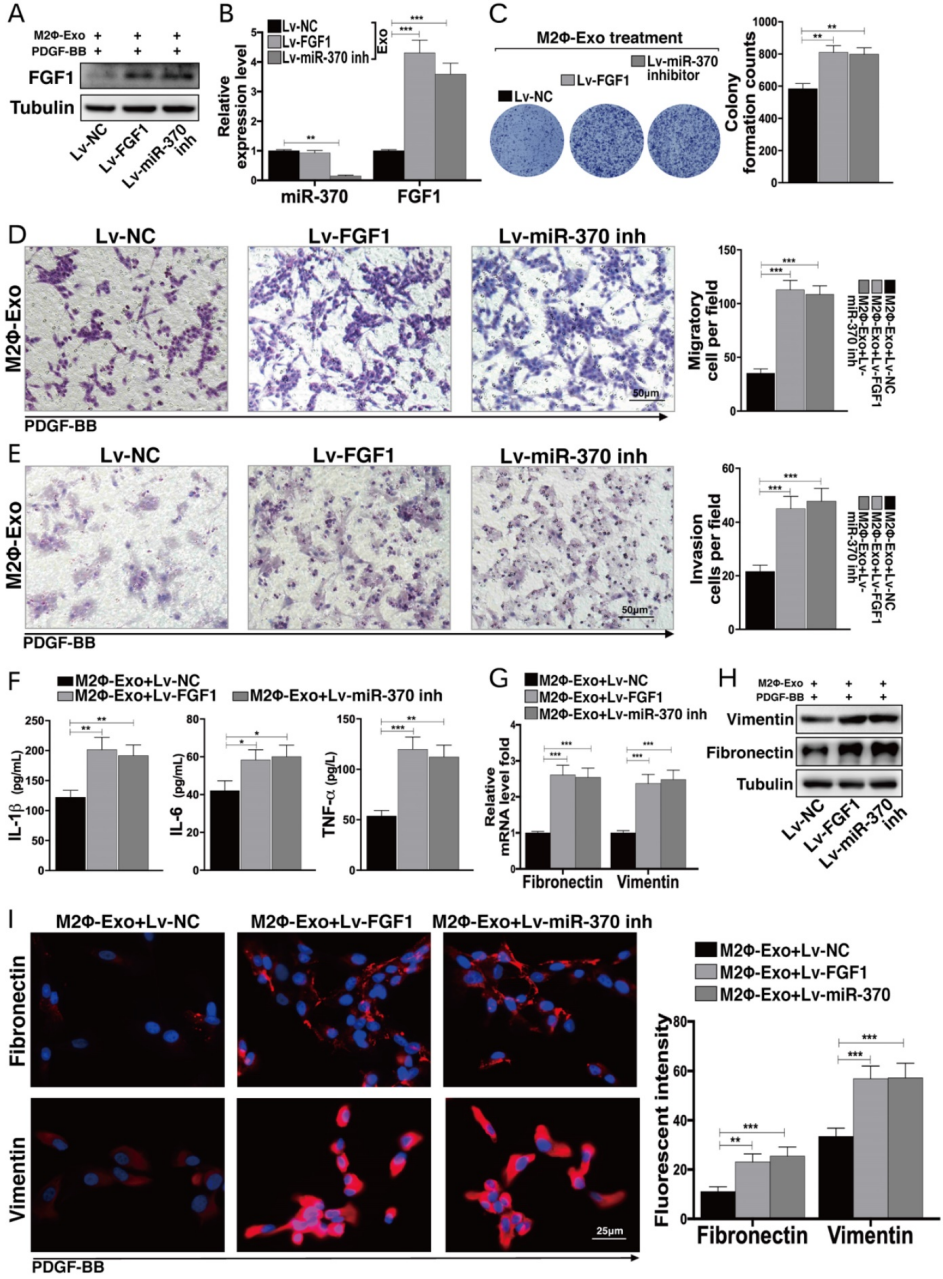
** Lv-FGF1 or Lv-miR-370 inhibitor inhibits the protective roles of M2Φ-Exos in PDGF-BB-treated ASMCs.** Lv-FGF1 or Lv-miR-370 inhibitor was further administrated in OVA- and M2Φ-Exos-treated ASMCs, (A) the protein expression of FGF1 and (B) miR-370 and FGF1 mRNA expression in ASMCs determined by Western blot and RT-qPCR, respectively. (C) Colonies of ASMCs measured by cell colony formation assay. (D) Migration and (E) invasion abilities of ASMCs measured by Transwell assays. (F) Expression of IL-1β, IL-6 and TNF-α in ASMCs determined by ELISA kits. (G) Expression of mRNA of fibronectin and vimentin in ASMCs determined by RT-qPCR. (H-I) Expression of fibronectin and vimentin in ASMCs determined by Western blot and immunofluorescence staining, respectively. Data were expressed as mean SD from at least three independent experiments. In panels B, C, D, E and F, data were analyzed by one-way ANOVA, while data in panels G and I were analyzed by two-way ANOVA, followed by Tukey's multiple comparison test. **p < 0.01, ***p < 0.001.

Likewise, Lv-FGF1 or Lv-miR-370 inhibitor was further administrated in PDGF-BB and M2Φ-Exos treated ASMCs, and the expression efficacy was determined by Western blot and RT-qPCR (Fig. 5A,5B). In PDGF-BB treated ASMCs, it was found that the number of cell colonies was increased upon FGF1 upregulation or miR-370 inhibition (Fig. 5C). In addition, the migration and invasion abilities of ASMCs were enhanced as well (Fig. 5D,5E). Besides, the secretion of IL-1β, IL-6 and TNF-α in ASMCs were increased (Fig. 5F). Moreover, the expressions of fibronectin and vimentin suppressed by M2Φ-Exos were increased after the further administration of Lv-FGF1 or Lv-miR-370 inhibitor (Fig. 5G-I).

### FGF1 activates the MAPK/STAT1 signaling pathway

**Figure 6:**
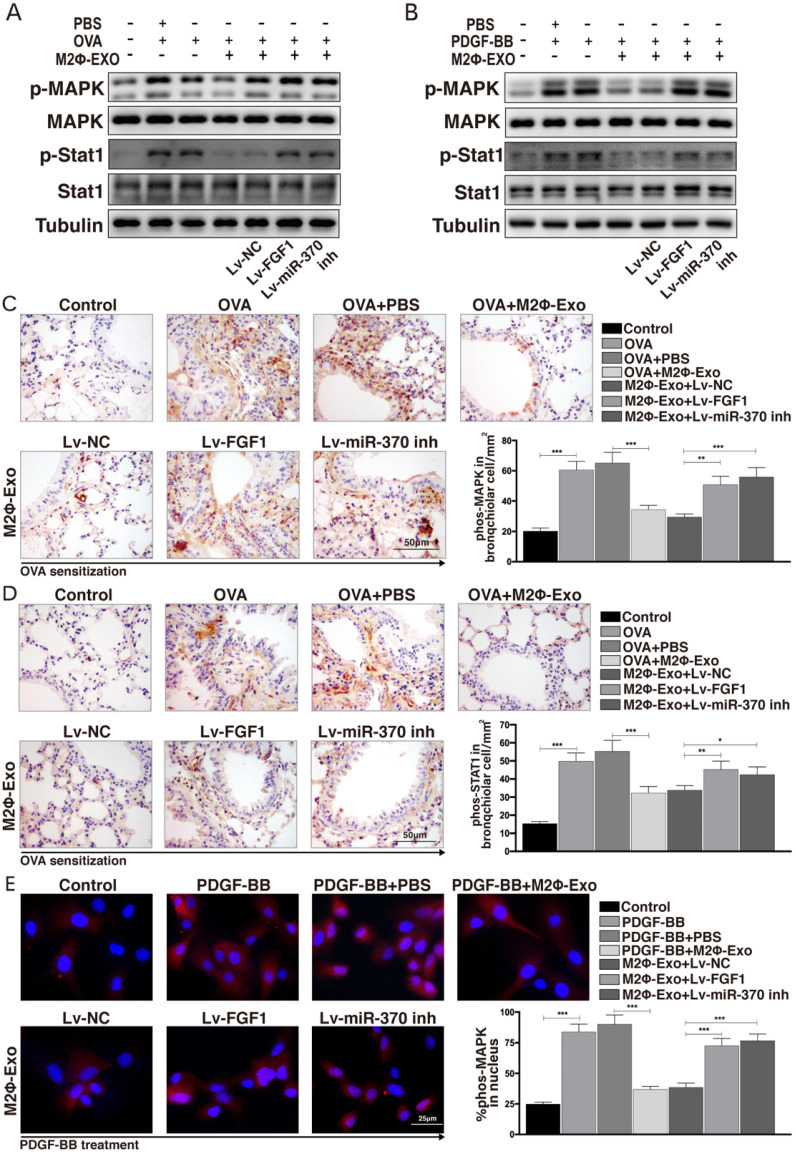
** FGF1 activates the MAPK/STAT1 signaling pathway**. (A) Phosphorylation levels of MAPK and STAT1 in mouse lung tissues determined by Western blot. (B) Phosphorylation levels of MAPK and STAT1 in ASMCs determined by Western blot. (C-D) Expression of p-MAPK and p-STAT1 in mouse lung tissues determined by IHC staining. (E) Nuclear translocation of p-MAPK measured by immunofluorescence staining. Data were expressed as mean SD from at least three independent experiments. In panels C and D, data were analyzed by one-way ANOVA, while data in panels E were analyzed by two-way ANOVA, followed by Tukey's multiple comparison test. **p < 0.01, ***p < 0.001.

A previous study noted that FGF1 played important roles in the progression of idiopathic pulmonary fibrosis through activating the MAPK signaling pathway 23, which attracted our attention that whether this signaling pathway is also implicated in asthma development. Thereby, we detected MAPK/STAT1 expression in OVA-induced mice using Western blot analysis and found that the phosphorylation levels of MAPK and STAT1 in mouse lung tissues were increased after OVA treatment, but then reduced by M2Φ-Exos administration; however, this reduction was further blocked by Lv-FGF1 or Lv-miR-370 inhibitor (Fig. 6A). Similarly, the same trend was found in ASMCs that the phosphorylation of MAPK and STAT1 was increased in ASMCs after PDGF-BB treatment but suppressed by M2Φ-Exos; again, further downregulation of miR-370 or upregulation of FGF1 recovered the activation of this signaling pathway (Fig. 6B). Moreover, the mouse lung IHC staining presented same results of p-MAPK and p-STAT1 expression (Fig. 6C,6D). In addition, the ASMCs immunofluorescence staining results showed that the nuclear translocation of p-MAPK was increased by PDGF-BB but inhibited by M2Φ-Exos; while, the nuclear translocation of p-MAPK was promoted by Lv-FGF1 or Lv-miR-370 inhibitor (Fig. 6E).

## Discussion

Limited understanding in the disease mechanisms is the major obstacle to the development of novel treatments to asthma. The lung is a complex organ comprises a wide array of structural and immune cells within the parenchyma and airway; therefore, cell-cell communication is crucial and exosomes are promising tools in lung biology and function including in asthma progression 6,24. In the present study, our results suggested that M2Φ-Exos convey miR-370 to reduce cell apoptosis, lung fibrosis, granulocyte secretion, inflammation in OVA-induced mice, and suppress aberrant hyperplasia and inflammation in PDGF-BB-treated ASMCs, during which the suppression of FGF1 expression and inactivation of the MAPK/STAT1 are possibly implicated.

The abundantly existed exosomes have been well documented to exert key functions in multiple biological development including cancer progression 25,26, inflammation 8,27 and respiratory diseases 28-30. However, there is limited evidence concerning the function of exosomes from macrophage sources in asthma. Here, after identification of the collected M2Φ-Exos by the surface biomarkers CD9, CD63 and TSG101 24,31, we administrated these exosomes into OVA-induced mice and PDGF-BB-treated ASMCs. Then, it was found that the fibrosis in mouse lung tissues, the number of eosinophilic granulocytes, neutrophil granulocytes and lymphocytes, the number of apoptotic cells in lung tissues, the inflammatory cell infiltration and the secretion of IL-1β, IL-6, TNF-α and MCP-1 were reduced by M2Φ-Exos administration. Likewise, the aberrant hyperplasia, migration and invasion, as well as the production of fibrosis-related factors (fibronectin and vimentin) and secretion of inflammatory cytokines (IL-1β, IL-6 and TNF-α ) in ASMCs were reduced. Abundant existences of eosinophils, neutrophils and lymphocytes are crucial for airway inflammation, a hallmark event of asthma 5. As for ASMCs, their aberrant activity accelerates the secretion of an extracellular matrix protein periostin, which is a biomarker of type 2 inflammation and acts on fibroblasts to trigger airway remodeling, promote mucus secretion and recruit eosinophils 4. Collectively, these findings suggested that M2Φ-Exos exerted potent alleviating roles in lung injury and inflammation, and the airway remodeling processes.

Considering miRNAs play multiple roles in allergic diseases including asthma 32, and they are a major cargo of exosomes that are responsible for the versatile functions of the exosomes. We screened out all expressed miRNAs from M2Φ-Exos by using miRNA sequencing(miRNA-seq) and miR-370 was identified as the significantly upregulated miRNAs after M2Φ-Exos treatment by RT-qPCR results in mouse lung tissues and ASMCs. The miR-370 has been noted to be implicated in control of several respiratory diseases. For instance, it was found to suppress LPS-induced apoptosis of WI-38 cells and inflammation injury in acute pneumonia 12. A similar trend was found that downregulation of miR-370 promoted LPS-induced acute pneumonia in A549 cells 22. Intriguingly, miR-370 has been noted as a tumor suppressor in non-small cell lung cancer 33. More relevantly, miR-370 has potent anti-fibrotic effect in lung of a rattus norvegicus pulmonary silicosis model 34. Here in this study, our experiments found that miR-370 was poorly expressed in asthma-like mouse or AMSC models but then recovered after M2Φ-Exos treatment. Further, downregulation of miR-370 by Lv-miR-370 inhibitor in mice or ASMCs blocked the protective impacts of M2Φ-Exos on lung injury and inflammation as well as ASMCs activity, indicating that miR-370 was at least partially responsible for the alleviating roles of M2Φ-Exos in asthma. In addition, FGF1 was identified as a mRNA target of miR-370. Interestingly, FGF1 upregulation by the lncRNA TUG1/miR-590-5p axis was found to accelerate proliferation and migration of airway smooth muscle cells and the consequent asthma development 35. Again, the rescue experiments in this research showed that overexpression of FGF1 in OVA-induced mice and PDGF-BB-treated ASMCs diminished the functions of M2Φ-Exos. Importantly, activation of the downstream MAPK signaling pathway has been well documented to participate in the roles of FGF1 in wound healing in lung epithelium 16 vascular endothelial growth factor promotion 36, and idiopathic pulmonary fibrosis 23. Therefore, we further explored phosphorylation of MAPK and its downstream signaling STAT1 in animal and cell models. It was found that the activity of MAPK/STAT1 was increased in OVA treated mice and PDGF-BB treated ASMCs compared to the controls. Further, M2Φ-Exos inactivated the MAPK/STAT1 signaling pathway and suppressed the nuclear translocation of p-MAPK. Activation of MAPK/STAT1 has been noted to be implicated in the inflammation and airway remodeling in asthma 37,38. Interestingly, activation of this signaling was suggested to participate in the pro-fibrotic and pro-inflammatory roles of PDGF-BB 39. Taken together, inactivation of the MAPK/STAT1 is possibly involved in the protective events by M2Φ-Exos.

To sum up, these findings provide new understandings in the mechanism involved in asthma progression. The clinical application potential of exosomes and the exosomal miRNAs have provided new hopes in the management of human diseases 40-42. We hope the findings of this study may provide the theoretical basis for the potential clinical usage of M2Φ-Exos in the management of asthma.

## Conclusion

In summary, our studies evidenced that M2Φ-Exos could reduce lung injury and inflammation, and inhibit ASMCs proliferation and the airway remodeling through carrying miR-370 and suppressing the FGF1/MAPK/STAT1 axis.

## Supplementary Material

Supplementary figures and tables.
