# Supplementary material for: MicroRNA-370 carried by M2 macrophage-derived exosomes alleviates asthma progression through inhibiting the FGF1/MAPK/STAT1 axis
Source: Int J Biol Sci. 2021 Apr 23;17(7):1795–807. doi: 10.7150/ijbs.59715 (PMC8120458; doi:10.7150/ijbs.59715)
Supplement: Supplementary file 1 — Supplementary figures and tables. [file ijbsv17p1795s1.pdf]

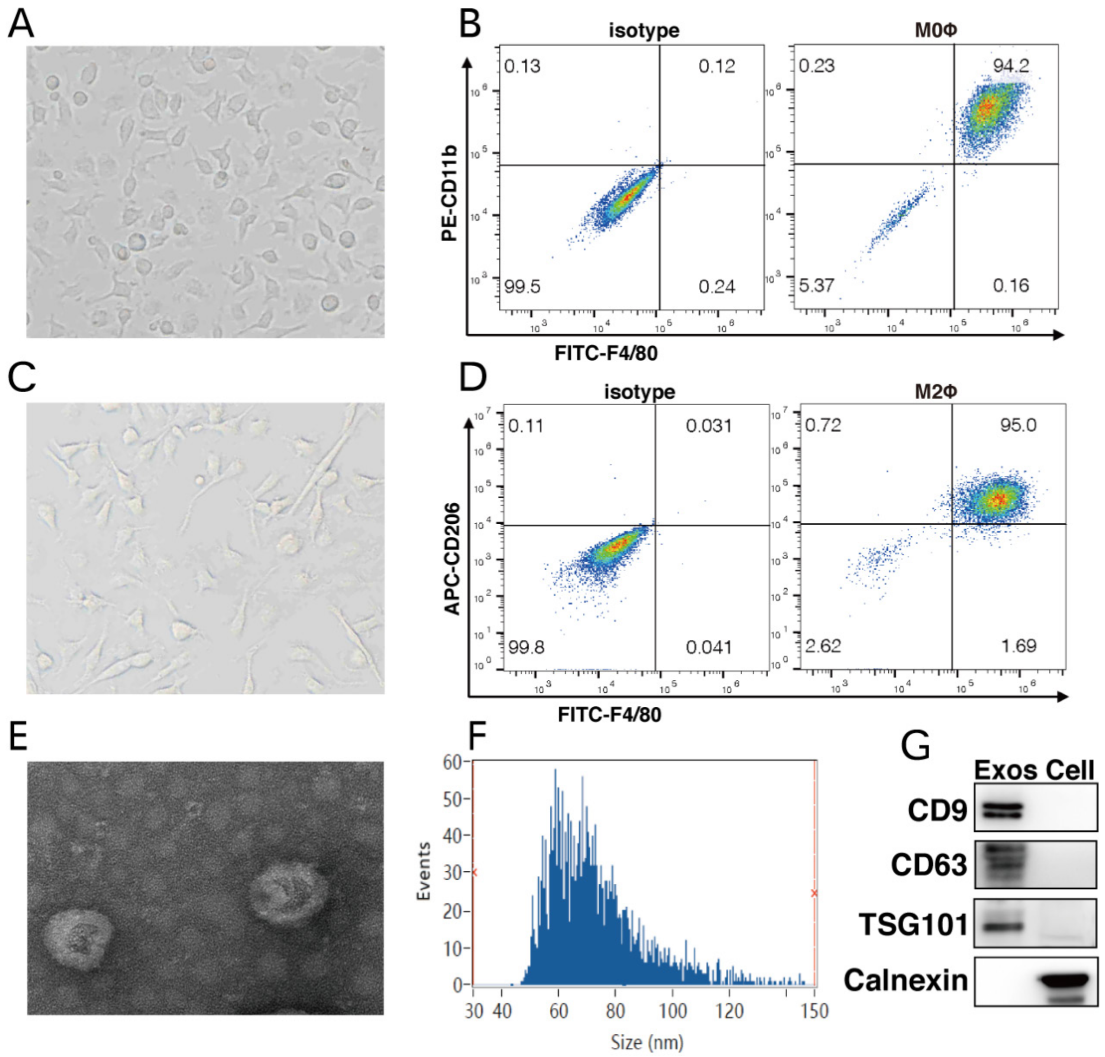

Fig. S1 Identification of M2 macrophages and the M2Φ-Exos. (A) Morphologic analysis of BMDMs under a microscope. (B) Expression of macrophage surface biomarkers CD11b and F4/80 determined by flow cytometry. (C) Morphologic analysis of BMDMs after IL-4 induction under a microscope.(D) M2 macrophage surface biomarkers CD206 determined by flow cytometry. (E) Shape of the particles evaluated under a TEM. (F) Particle size analyzed by nanoanalysis. (G) Expression of exosome marker proteins CD9, CD63 and TSG101 as well as cell marker protein Calnexin determined by Western Blot.

Table1. List of primers

Gene	Primer sequence(5'-3')
Fibronectin	F:ATGTGGACCCCTCCTGATAGT<br>R: GCCCAGTGATTTCAGCAAAGG
Vimentin	F:GCTGCGAGAGAAATTGCAGGA<br>R:CCACTTTCCGTTCAAGGTCAAG
FGF1	F: GGGGAGATCACAACCTTCGC<br>R: GTCCCTTGTCCCATCCACG
GAPDH	F: AGGTCGGTGTGAACGGATTTG<br>R: GGGGTCGTTGATGGCAACA
miR-370	F: CTGCTGGGGTGGAACCT<br>R: GAACATGTCTGCGTATCTC
U6	F:GTCTCCTCTGACTTCAACAGCG<br>R:ACCACCCTGTTGCTGTAGCCAA
